# Drug development in pediatric psychiatry: current status, future trends

**DOI:** 10.1186/1753-2000-6-7

**Published:** 2012-02-07

**Authors:** John S March, Joerg M Fegert

**Affiliations:** 1 Department of Psychiatry and Behavioral Sciences, Duke University Medical Center, Durham, North Carolina; 2 Duke Clinical Research Institute, Duke University Medical Center, Durham, North Carolina; 3 University Hospital Ulm, Department of Child and Adolescent Psychiatry/Psychotherapy, Steinhövelstr 5, 89075 Ulm, Germany

## Introduction

Reflecting the fact that regulatory agencies recently required companies to initiate a pediatric drug development plan earlier in the drug development sequence for compounds first developed for adults, most psychiatric drugs for children still remain the offspring of adult drug development programs, viz., except for the psychostimulants, very few psychiatric medications have been developed for children and adolescents by first intent [1]. However, two irreversible trends are gradually shifting intervention development for psychiatric disorders away from a focus on adult organisms to a focus on developing organisms. First, epidemiological data indicate that the great majority of mentally ill adults were first mentally ill as children [2], and that this effect is evident as early as two years of age [3]. Second, recent advances in translational developmental neuroscience have shown that mental illness of all types can be referenced directly to the developing central nervous system and its interactions with the environment [4]. The knowledge that mental disorders are early onset, trajectory-based brain illnesses has enormous implications for the nature and organization of how we understand interventions for psychiatric patients of all ages [5,6]. Put succinctly, to preempt, prevent and cure psychiatric disorders, it will be necessary to translate insights about molecular pathways for mental illness into druggable targets that directly reflect key neurodevelopmental processes that form trajectories of atypical as contrasted to typical development [7]. To this end, this commentary will describe drug development in pediatric psychiatry with reference to three converging perspectives: fundamental biology and target identification, early phase clinical pharmacology, and the importance of biomarkers in the shift to personalized medicine.

## Discussion

### Fundamental Biology and Target Identification

As shown in Table 1 drug development ideally follows a series of well-recognized steps: understanding disease-specific cellular and molecular pathobiology; target identification and assay development; identification and optimization of a lead molecule; toxicology and manufacturing; and eventually a series of investigational new drug (IND) trials that are expected to lead to a successful New Drug Application (NDA). While there are a variety of incentives and requirements that differentiate pediatric from adult drug development in psychiatry (for reviews, see [8,9]), the key point for our purposes is that a rich understanding of molecular driver (primary to off trajectory development) and modulatory pathways will reveal potentially druggable targets as well as diagnostic and response biomarkers that will proceed through the drug development process together. In a striking example from cancer biology, gefitinib, a biologic (an antibody as contrasted to a small molecule) that antagonizes the receptor for epidermal growth factor (EGFR), is a potent treatment for non-small cell lung cancer (NSCLC), but only in those patients with a particular genetic form of EGFR [10]. In the cancer model, a small number of commonly mutated gene "mountains" and a much larger number of gene "hills" that are mutated at low frequency comprise the driver pathways for oncogenesis [11,12]. If neuropsychiatric disorders follow the cancer model--and it appears from studies in autism, schizophrenia, bipolar disorder and epilepsy that this may be the case [13-15]--progress in understanding the nature and heterogeneity of complex psychiatric diseases should presage the development of targeted disease modifying treatments for psychiatric disorders [16].

**Table 1:** Steps in Drug Development

*Discovery and Concept Development*
• Target Identification and Validation

• Assay Development and Screening

• Lead Molecule Discovery

• Lead Molecule Optimization

• Pharmacodynamics and Toxicology

*Proof of Concept/IND*

• Clinical Phase I: PK/PD, ADME, abuse liability

• Phase II Proof of Concept

*Product Development*

• Phase III Registration

• NDA Approval

• Phase IV Post Marketing

Until psychiatric genetics yields validated targets or even clinically efficacious stratification markers for the major classes of psychiatric illness [17], the approach to drug development in psychiatry will remain where it always has been, namely opportunistic rather than mechanistic. In the past and to some extent today, drug development in psychiatry deviated from step one in that serendipity usually based on clinical observation lead to small proof of principle trials, animal studies, and then to rational medical chemistry efforts [18], viz. the progression from imipramine to fluoxetine. In fact, other than lithium [19], many if not most current generation antipsychotics and antidepressants have chlorpromazine as the root molecule, with modifications based on clinical insights like the fact that chlorpromazine itself has antidepressant as well as antipsychotic effects [20]. Some argue that opportunistic insights will remain the primary route for new medicines in psychiatry for the next decade or more [18]; others claim that the tremendous investment in fundamental biology on the part of industry and the NIH shortly will begin to pay off in new, innovative, disease state therapeutics [21]. In favor of the latter are newly identified therapeutics that target three mechanistically distinct glutamate pathways: First, intraveneous ketamine produces promising and rapid antidepressant effects in treatment resistant depression (for review see [22]). This finding, which seems to involve rapid effects on synaptogenesis presumably via brain derived neurotrophic factor (BDNF), TrkB and mTOR pathways [23], has generated considerable interest in orally available N-methyl-D-aspartate antagonists and AMPA receptor potentiators as treatments for depression [24]. Second, selective agonists for metabotropic glutamate 2/3 (mGlu2/3) receptors that show antipsychotic potential in animal models of schizophrenia [25] show promise in clinical trials in patients with schizophrenia [26]. And third, genetic insights into the molecular pathobiology of Fragile X syndrome have lead to the development of a class of compounds, the mGluR5 antagonists, that reverse the Fragile X phenotype in animal models of Fragile X [27]. Trials in humans are just now getting underway [27,28]. If successful, Fragile X will be the first psychiatric disorder in which a potentially curative treatment was developed mechanistically from gene identification to pathophysiology in animal models to novel therapeutics in humans.

**Figure 1:**
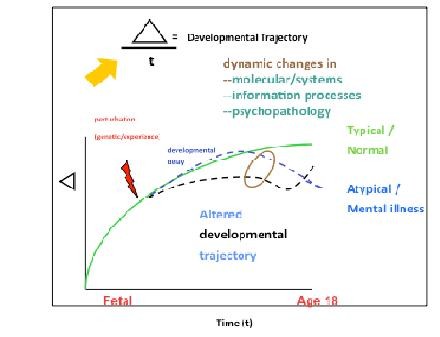
**Translational Developmental Neuroscience**. depicts the time course of atypical versus typical development. The red arrow at in early childhood indicates a perturbation followed by an immediate or later onset trajectory deviation involving dynamic changes in molecular systems, information processes running on hierarchically distributed neural networks, and resulting psychopathology, which when sufficiently altered (brown circle) comes to clinical attention. Opportunities for preemption predate the onset of clinical illness either before or early in the prodromal period of delayed development.

For neurodevelopmental targets--the only possible targets that are preemptive--that exhibit complex rather than Mendelian genetics, the identification of personalized drugable targets on driver pathways is particularly challenging [4,29,30]. As shown in Figure 1, the processes that go awry in mental illness likely involve time-sensitive modulation in gene expression, cellular interactions, circuit formation and function, and behavior, all interacting alongside environmental experience to produce typical or atypical developmental trajectories [31,32]. Moreover, the onset of symptoms may not indicate the actual beginning of the illness, e.g. symptoms may appear long after the causal processes leading to mental illness have begun (see, for example, DISC1 and schizophrenia [13]). Acute or downstream, these processes necessarily will become the targets for interventions that aim to restore normal developmental process or to initiate compensatory processes that return a patient to a functional neurodevelopmental trajectory [7,33]. In each case, points of leverage (drugable targets) utilizing small molecules, biologics, RNA or protein aptamers targeting pre- or post-synaptic receptors or intracellular signal transduction pathways will only emerge when the relevant disease-specific molecular pathways are clarified allowing disease relevant targets to be distinguished from relatively common pathways that are involved in basic cell functions and, thus, in both normal and disease states, viz. the mTOR pathway in autism [34]. Despite the difficulty of the task--and it is indeed humbling--the availability of personal genomic and a wealth of large-scale biological datasets provide an unprecedented opportunity to identify therapeutically relevant targets that will be both druggable and disease modifying [35].

Emphasizing the importance of developmental neuroscience to understanding the fundamental biology of mental illness, a 2008 National Advisory Mental Health Council (NAMHC) Workgroup (co-chaired by John March and Pat Levitt) issued a report entitled *Transformative Neurodevelopmental Research In Mental Illness *that strongly recommended that the NIMH refocus its discovery and translational neuroscience portfiolio on identifying and translating testable developmental targets into new preemption and treatment efforts. On the other hand, while tools and technologies, such as the ability to define the patterns of gene expression and manipulate the major pathways for signal transduction in brain subregions as they impact early development, now permit interrogating the CNS in model organisms [36] or in induced pluripotent stem cells [37], the translational payoff in preventive pediatric indications is years and perhaps even decades away [38-40]. In the meantime, as illustrated by the attempts to intervene in prodromal schizophrenia [41,42], improved disease state therapeutics that involve early intervention in the illness prodrome or early in its course will increasingly come to dominate psychiatric therapeutics.

### The Shift to Early Phase Clinical Pharmacology

In addition to the promising impact of improved target identification, the shift toward early phase clinical pharmacology, is also being driven by a variety of other factors, including high costs, an empty pipeline, success rates that are lower for neuroscience trials that in any other therapeutic area, competition with generics, and the need to satisfy not only the FDA but payers regarding a treatments incremental value. Consequently, some companies have pulled out of psychiatry R&D altogether [36,43] and others are downsizing, preferring to wait until improvement in our understanding of fundamental biology generates novel drugable targets that can be move through the preclinical drug development process and eventually into early phase clinical trials programs [21]. Given the need to substantially increase the number and quality of innovative, cost-effective new medicines without incurring unsustainable R&D costs, Paul and colleagues recently recommended a shift in emphasis from large and expensive Phase III programs to a focus on multiple "quick win, quick fail" proof of concept (POC) trials [43]. The central idea is to promote to Phase III only those compounds that have a high probability of success as indicated by the results of a carefully executed Phase II program.

Since the impetus for developing novel therapeutics is frequently dependent on work in academic laboratories or in biotech or small pharma "spinoffs" from academia [44,45], it is likely that the development process for new molecules that enter POC increasingly will depend on collaborations between industry, the NIH and academic medical centers [21,46,47]. In this context, Tom Insel, the current NIMH Director, recently articulated a strategic plan for the NIMH that emphasizes the importance of novel interventions that emerge from the NIMH's investment in discovery and translational neuroscience [6]. The enduring vision is to explicate the underlying neurobiology, identify new treatment targets, develop drugs, biologics, devices and refined psychosocial interventions for new targets, and do so in a lifespan context that emphasizes early developmental events and is personalized. To lay the foundation for developing the next generation of interventions for mental disorders, especially those interventions that are tailored to the individual (i.e., that are personalized) and that prevent the damaging consequences of these illnesses (i.e., that are preemptive), the NAMHC (David Lewis and John March, co-chairs) recently issued a report, *From Discovery to Cure *[48], that provides explicit guidance regarding promising research investments and strategies. Capitalizing on key NIH investments in technologies for the development of novel therapeutics [21,47,49], the workgroup reports puts in place a smooth and efficient process for intervention discovery, from pre-clinical studies to Phase I safety and dose finding studies in typical humans through proof-of-concept Phase II studies, and the establishment of clinical efficacy. Consequently, the NIMH also is moving away from studying current generation treatments and toward early phase intervention development [48]. Importantly, while pediatric drug development programs will continue to follow adult intervention development [29], the NIMH is now turning toward building a knowledge environment in which mentally ill youth are viewed as a key target population.

### Biomarkers and the Shift to Personalized Medicine

Biomarkers, which emerge from the process of target identification, are the foundation of stratified and eventually personalized diagnosis and treatment. Stratified medicine means using biomarkers to tailor health care to a group of patients with similar characteristics; personalized medicine refers to using biomarkers to tailor health care to the needs of the individual patient. According to a recent IOM report on neuroscience biomarkers[50], biomarkers are clinically applicable quantitative measurements about biological processes, a disease state, or about response to treatment. A biosignature is a collection of biomarkers optimized for predictive validity. "-Omics" biomarkers refer to the contribution of genes, proteins, and metabolic pathways to human physiology and to the fact that variations along -omics pathways are thought to lead to disease vulnerability. Specific -omics technologies (often called platforms) include genetics/genomics, epigenetics, transciptomics, proteomics and metabolomics. Neuroimaging biomarkers include MRI, PET, QEEG and MEG, and can be combined with -omics markers to increase precision in systems biological approach that examines the interactions between the diverse aspects of biological systems as they give rise to organismal behaviors in health and in diseases [51,52]. A biomarker generally provides one of two kinds of information: diagnostic or therapeutic. Using FDA/industry terminology therapeutic biomarkers are referred to as companion (to treatment) diagnostics that do one of the following: stratify patients on choice of treatment, tailor the dose of treatment, predict response early in treatment, or provide a surrogate endpoint to facilitate the study of intervention efficacy.

While the FDA has released guidance documents on validating biomarker/biosignatures,[53,54] there is still considerable confusion in our field over what is required to identify, validate and apply a new test in clinical settings[55,56] so much so that the AACAP Research Forum at the 2011 annual meeting in Toronto was devoted to explicating biomarker sciences. In an innovative example of biomarker based therapeutics in a public-private partnership framework [57], the Foundation for the NIH (FNIH) is sponsoring biomarker stratified adaptive RCTs in advanced breast cancer, the Investigation of Serial Studies to Predict Your Therapeutic Response (I-SPY) studies, that use the patient's own tumor tissue and a commercially available gene chip, the MammaChip, to improve treatment outcomes in by using a companion diagnostic to identify the best treatment strategy at each point in disease progression, e.g. to apply stratified medical strategies to personalize care [58]. In psychiatry, Alzheimer's disease provides a very useful model for understanding how to think about early intervention for a trajectory-based neurodevelopmental disorder [59]. In a series of papers [60-62], the Alzheimer's Disease Neuroimaging Initiative (ADNI) with funding from the FNIH and industry has promoted the development of CSF proteomic, fMRI and PET biomarkers that accurately track the progression from normal elderly to mild cognitive impairment (MCI) to Alzheimer's disease (AD). Understanding the progression of AD biology over time has enabled the identification of disease and response biomarkers as well as surrogate endpoints for clinical trials. In turn, this has facilitated the development of disease-modifying treatments like the humanized anti-Abeta monoclonal antibody, bapineuzumab, that is currently undergoing Phase III clinical trials [63]). The point here is not that bapineuzumab by itself is going to transform the lives of patients with Alzheimer's Disease--it faces a variety of hurdles [64]-but rather that the availability of, for example, a PET biomarker allows medicines to be given much earlier in the disease course thereby offering the potential of disease modification. Given the obvious parallels, we might expect that conceptual models derived from AD should positively influence drug development programs for neurodevelopmental disorders at the other end of the age spectrum.

### Ethical considerations and need for new trial regulations

Future psychopharmacological interventions will aim at identifying targets to predict distal outcomes. The identification of a certain risk in the context - for example of a screening procedure in early childhood - might lead to preventive therapeutic interventions to prevent, for example, schizophrenia [42], or even Alzheimer's Disease [65]. Psychiatrists now try to describe schizophrenia prodrome as a disorder based on descriptive symptoms. If schizophrenia prodrome would be introduced as a disorder, on intervening in prodromal schizophrenia this disorder could be an indication in the context of current drug regulations. In McGorry's seminal study of intervening in the adolescent-onset schizophrenia prodrome [66], only some of the patients described as at risk for schizophrenia eventually developed schizophrenia. But still the number needed to prevent could be studied in the context of a trial, only as McGorry notes if the risk-balance equation is in the proper direction [67]. At the moment there is no legal framework for such a type of preventive intervention and there is relatively little ethical debate about these issues [68]. In child and adolescent psychiatry the recent debate on early preventive interventions in the prodromal phase of schizophrenia might serve as an example [69]. As at the moment there is no procedure to label medications for preventive intervention except from vaccines.

When talking about statistically relevant risks to develop late onset neuropsychiatric diseases no controlled trial can be imagined studying the real outcomes of an intervention. The current regulations and procedures for clinical trial that are well established are inadequate in this context [70]. Therefore the identification of molecular targets etiologically responsible for the outcome is important. For surrogate endpoints to be applicable to preemptive strategies, their predictive validity both positive and negative relative to distal clinical outcomes must to sufficiently robust to be ethical [71]. It also is unclear, who can make informed decisions about early interventions in children concerning their adult life. In most legal systems parents are thought to be best decision makers because usually they try to consider the best interests of the child, but it is well known, that in the aim of granting their children the best possible treatment, parents may act in an over-protective or over-interventional manner. The ethical judgement seems to be relatively obvious in monogenetic disorders, such as Fragile X syndrome where MGluR 5 antagonists are promising disease-modifying therapies [27]. Here a clear diagnosis allows to predict severe consequences and even if the phenomenology of the disorder is not yet visible there is a 100% risk for a later development of the full blown disease. In this context, it is clear that the persons carrying this genetic information can be called ill at birth or even before birth and the ethics of treatment discussed rationally in an ethical context. But what about relative risks, if screening tests discovers a 30% elevation over the normal risks to develop a disease like depression? Will parents be able to decide that it is reasonable to intervene to prevent a relatively smaller risk and even more so what parents will allow testing of new substances and concepts in their children? It becomes clear that the severity of the outcome and the identifiable costs of a disease, perhaps defined by reduced quality of life years (QALY) or similar measure, will play an important role in the ethical judgement. As the field moves toward preemptive interventions, it is our obligation as clinicians and clinical neuroscientists to introduce and to reinforce that debate in our discussions with researchers from fundamental research usually dealing with cells or animals. As advances in fundamental biology fuel the development of potentially preemptive interventions for children by first intent, the discussion of ethical will need to begin in the preclinical translational space before compounds enter Phase I and proof-of-concept trials.

## Conclusions

As described in a recent article by the NIMH Director, Tom Insel, on transforming psychiatry as a clinical discipline [29], the age of symptomatic diagnosis and current generation treatments is passing; the age of interventions that emerge from the revolution in translational developmental neuroscience has begun. The twin NIMH Council workgroup reports on translational developmental neuroscience [5] and interventions research [48], respectively, which shift the National Institute of Mental Health away from current generation treatments and toward early phase clinical pharmacology, presage the development of just these kind of preemptive treatments. Because these newer interventions will emerge from an improved understand of the fundamental biology of the illnesses, they should be more effective in patients who are ill and, excitingly, will eventually become preventive if not preemptive, e.g. they will be delivered to very young children who are at risk but not yet showing early signs of mental illness. As a result, pediatric psychiatry will increasingly become the front end (the most important end) of a lifespan developmental model for mental illnesses. More than a little humility is required as this vision unfolds over the next many years. For a while, studies in adults will still lead studies in youth: developing interventions for mentally ill youth will emerge once the fundamental biology catches up such that science drives innovation and innovation drives application in the form of interventions. As part of this process, biomarkers on the road to stratified and ultimately personalized medicine will be a key development--finally, the age of molecular diagnosis and the dawn of the age of companion diagnostics to optimize treatment for psychiatric illness. For the field of pediatric psychopharmacology to thrive it will be important to embrace and actively participate in this revolution, including addressing its ethical implications, so that mentally ill youth are viewed as a key target population and, consequently, truly preemptive, preventive and curative interventions will be developed for children by first intent [1,8,9].

## Conflict of interest statement

Dr. March has served as a consultant or scientific advisor to Pfizer, Lilly, Avenir, Alkiermes, Atentive, BMS, Johnson and Johnson, MedAvante, Otsuka, Psymetrix, Scion, Shire, Travena, Vivus, and Widay Pharmaceuticals; has received research support from Eli Lilly and Pfizer; has received study drug for an NIMH-funded study from Eli Lilly and from Pfizer; is an equity holder in MedAvante; receives royalties from Guilford Press, Oxford University Press and MultiHealth Systems; and receives research support from NARSAD, NIMH and NIDA. As a member of the National Advisory Mental Health Council, Dr. March co-chaired the workgroups on *Tranformative Neurodevelopmental Research in Mental Illness *and *From Discovery to Cure*. Dr. March has not engaged in industry promotional work, e.g., speakers bureau or training, for over 15 years. Dr. Fegert has received research support from the Eli Lilly Foundation, Janssen Cilag, Boehringer Ingelheim and from Celltech/USB. Further research support from the German Ministries for Family Affairs, Senior Citizens, Women and Youth, and for Education and Research (BMFFSJ, BMBF), the Volkswagen foundation, Schweizer Bundesamt für Justiz, the Deutsche Forschungsgemeinschaft and various non-profit organizations. He is consultant or advisor for Janssen Cilag, Servier, Aventis Bayer, Bristol-MS, J&J, Celltech/USB, Eli Lilly, Medice, Novartis, Pfizer, Lundbeck, Sanofi-Synthelabo, VFA & Generikaverband, he received travel support from the Vatican, NIMH, AACAP, DFG, EU and European Academy and several non profit-organizations.
